# Supplementary material for: Prolonged photo-carriers generated in a massive-and-anisotropic Dirac material
Source: Sci Rep. 2018 Jun 13;8:9073. doi: 10.1038/s41598-018-27133-6 (PMC5998121; doi:10.1038/s41598-018-27133-6)
Supplement: Supplementary file 1 — Supplemental Material [file 41598_2018_27133_MOESM1_ESM.pdf]

Supplemental Material: Prolonged photo-carriers generated in a massive-and-anisotropic Dirac material

Munisa Nurmamat[1*], Yukiaki Ishida[2], Ryohei Yori[1], Kazuki Sumida[1], Siyuan Zhu[1], Masashi Nakatake[3], Yoshifumi Ueda[4], Masaki Taniguchi[4], Shik Shin[2], Yuichi Akahama[5], and Akio Kimura[1¶]

[1]Department of Physical Sciences, Graduate School of Science, Hiroshima University, 1-3-1 Kagamiyama, Higashi-Hiroshima 739-8526, Japan
[2]Institute for Solid State Physics, the University of Tokyo, 5-1-5 Kashiwa-no-ha, Kashiwa, Chiba 277-8581, Japan
[3]Aichi Synchrotron Radiation Center, Aichi Science & Technology Foundation, 250-3 Minamiyamaguchi-cho, Seto 489-0965, Japan
[4]Hiroshima Synchrotron Radiation Center, Hiroshima University, 2-313 Kagamiyama, Higashi-Hiroshima 739-0046, Japan
[5]Graduate School of Material Science, University of Hyogo, 3-2-1 Kouto, Kamigori-cho, Ako-gun, Hyogo, Japan
*e-mail: munisa627@hiroshima-u.ac.jp ¶e-mail: akiok@hiroshima-u.ac.jp

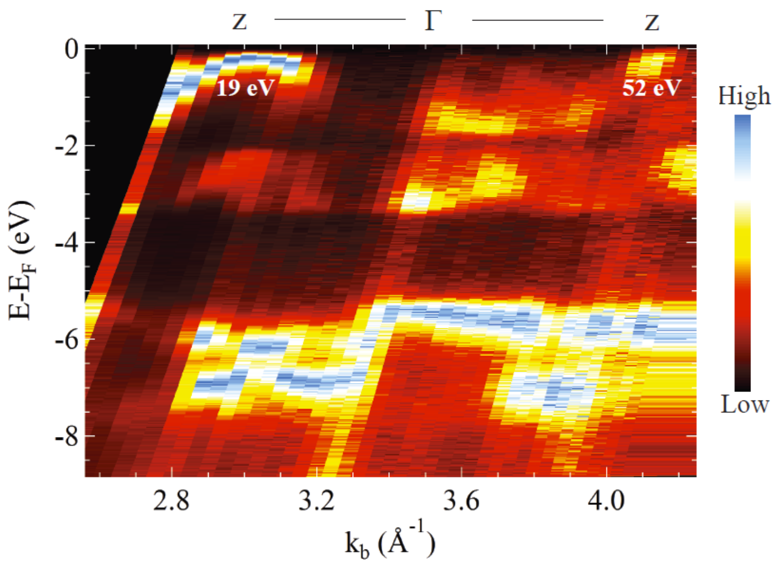

Figure S1. Energy band dispersion of BP along Z-Γ-Z direction (*b*-axis) using photon energy *hν* =19-56 eV. Valence band maximum can be seen at photon energy of 19 eV and 56 eV.

We have carefully checked the stability of BP surface by X-ray photoelectron spectroscopy (XPS) during the measurements. One could see the very clear $2p_{3/2}$ and $2p_{1/2}$ doublets which are characteristic of crystalline BP near the 130 eV from the XPS spectrum without any oxidized signature as shown in the supplementary Figure 2.

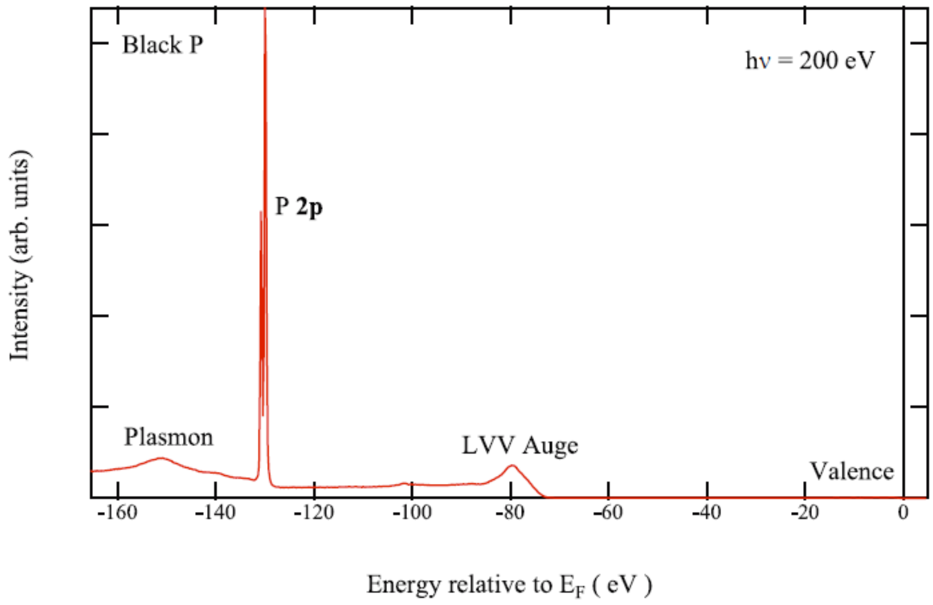

Figure S2. High-resolution P 2p core level acquired on *in situ* cleaved BP surface with photon energy of 200 eV.

In order to provide the spectral function of the system and strengthen the interpretation of the XPS spectra, we also show the wide range ARPES spectrum [see supplementary Figure 3] and we do not find any signal from Oxygen 2p state around 6 eV binding energy. Furthermore, samples we used here have been *in situ* cleaved, and all the measurement taken in an ultrahigh vacuum, which can protect samples from oxidization. Considering above, we conclude our sample is free from oxidation during the measurement.

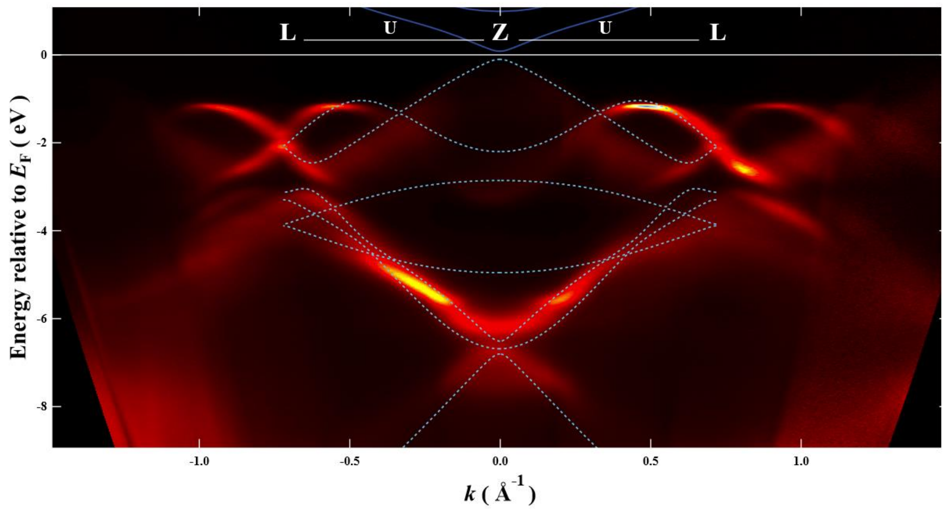

Figure S3. Valence band dispersion along the Γ-X direction with showing wide range taken at photon energy of 26 eV. Dashed line indicates the correspondent band dispersion by using first principle calculation. There is a good agreement between experimental and calculation, again signifies no oxidation feature of BP surface.
